# Antioxidant Interactions between Major Phenolic Compounds Found in ‘Ataulfo’ Mango Pulp: Chlorogenic, Gallic, Protocatechuic and Vanillic Acids

**DOI:** 10.3390/molecules171112657

**Published:** 2012-10-26

**Authors:** Hugo Palafox-Carlos, Joana Gil-Chávez, Rogerio R. Sotelo-Mundo, Jacek Namiesnik, Shela Gorinstein, Gustavo A. González-Aguilar

**Affiliations:** 1 Research Center for Food & Development, A.C. (CIAD), Carretera a Ejido La Victoria Km 0.6, Hermosillo Sonora 83304, Mexico; 2 Research Department of Polymers & Material Sciences (DIPM), Universidad de Sonora, Calle Rosales y Blvd. Luis Encinas s/n, Col. Centro, P.O. Box 130, Hermosillo Sonora 83000, Mexico; 3 Department of Analytical Chemistry, Chemical Faculty, Gdańsk University of Technology, Gdańsk 80952, Poland; 4 The Institute for Drug Research, School of Pharmacy, The Hebrew University, Hadassah Medical School, Jerusalem 91120, Israel

**Keywords:** mango, antioxidants, phenolic acids, interactions

## Abstract

Phenolic compounds are known to have antioxidant capacity; however, there is little information about molecular interactions between particular phenolics found in fruits at different developmental stages. Therefore, the total antioxidant capacity of the phenolic compounds of a fruit may not correspond to the sum of individual antioxidant capacity given by antioxidants from that tissue. In this study, individual antioxidant capacity and the interactions of four major phenolic compounds (chlorogenic, gallic, protocatechuic and vanillic acid) found in ‘Ataulfo’ mango pulp were tested using the DPPH assay. Significant synergism was found in the majority of the all combinations, as well as the combination of the four phenolics. However, antagonism was also observed between some molecules. This work demonstrated particular interactions that may occur in a complex environment within the complex framework of a natural food. The present results may also assist in the future design of functional foods or ingredients based on their antioxidant activity and their synergistic or antagonist interactions.

## 1. Introduction

Phenolic acids are antioxidant molecules that are in the limelight of clinical and epidemiological research because their demonstrated value as the antioxidant components of fruits and vegetables [1]. These foods also contain a wide variety of antioxidant bioactive compounds (carotenoids, vitamins, among others) that provide health benefits to consumers [2,3,4,5]. Mango (*Mangifera indica* L.) fruit is an excellent source of dietary antioxidants, such as ascorbic acid, carotenoids, and especially phenolic compounds [6]. The health benefits have been demonstrated *in vivo* because of their remarkable antioxidant capacity (AOXC) [7,8].

Recently, it was reported that mango ‘Ataulfo’ had the highest phenolic content and AOXC among several mango cultivars [9]. According to Palafox-Carlos *et al*. [10], the major phenolic compounds found in ‘Ataulfo’ mango pulp are chlorogenic, gallic, protocatechuic and vanillic acid. Consumption of these phenolic acids has been found to have an inverse relationship with the incidence of various diseases and chlorogenic and gallic acids may be closely related to those benefits for consumers [9]. 

The relationship between phenolic bioactive compounds, their AOXC [11] and the health benefits are well established. However, information about phenolic acids and their interactions on the AOXC is scarce. A previous study reported individual phenolic changes during ripening and affected to different extent the AOXC in durian (*Durio* sp.) fruit [5]. 

Each phenolic compound has a different AOXC depending on its structure, number of aromatic and hydroxyl groups and their distribution in the structure [12,13]. In foodstuffs the composition of bioactive phenolics is complex and it is assumed that all account to the overall AOXC [14]. However, interactions between phenolics could be happening and they could be additive, synergistic or even antagonistic. To gain further insights about interactions in mango pulp, we sought to evaluate the individual and combined antioxidant activities of the four major phenolics in this fruit, in order to provide the bases towards rationally designed nutraceuticals.

## 2. Results and Discussion

**Figure 1:**
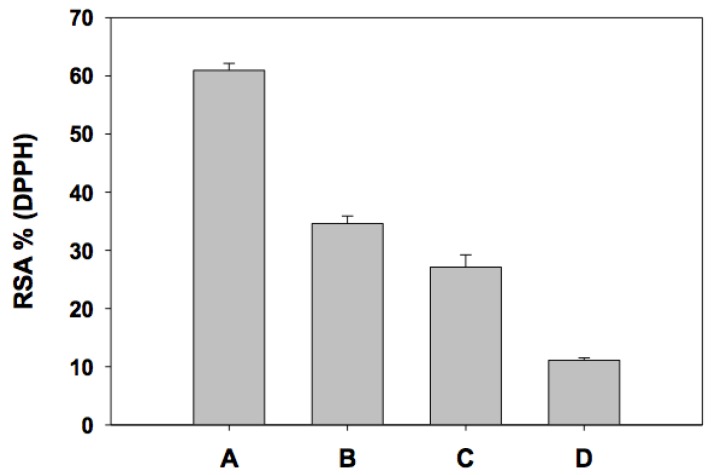
Individual antioxidant capacity of phenolic acids at 0.2 mM. Gallic acid (**A**), chlorogenic acid (**B**), protocatechuic acid (**C**) and vanillic acid (**D**).

The individual AOXC of the major phenolic compounds in mango ‘Ataulfo’ was determined and it is shown in Figure 1. The AOXC for each phenolic compound at determined at 0.2 mM. The assay was done using 2,2-diphenyl-1-picrylhydrazy (DPPH) radical and it is reported as percentage of radical scavenge capacity (RSA) (see Experimental section below).

**Figure 2:**
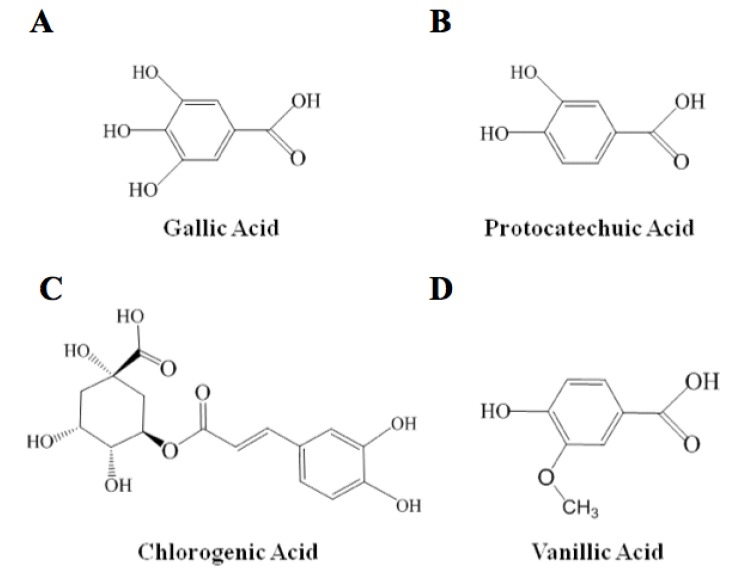
Chemical structure of the compounds studied in this work.

Gallic acid (A) had the highest antioxidant capacity with 61% RSA, followed by protocatechuic acid (B), 35% RSA; chlorogenic acid (C), 28% RSA and vanillic acid had the lowest value of 11% RSA. Our results are similar to those reported by Rice-Evans *et al*. [15], where gallic acid exhibited the highest AOXC, and vanillic acid the lowest among several phenolic compounds. In particular, phenolic acids are considered to be efficient hydrogen donors due to their characteristic carboxylic group, which is easily ionized [16]. Evaluating the individual antioxidant potential of phenolic compounds is a topic that has taken attention in our laboratory in order to understand real potential and biological action of phenolic antioxidants. In Figure 2 are shown the chemical structures of the four phenolic acids studied. When we compared the structures of phenols of one aromatic ring and the AOXC, the number of hydroxyl groups correlated positively with antioxidant capacity against DPPH. Thus gallic acid presented the highest antioxidant capacity and had four hydroxyls, followed by procatechiuic acid with two and vanillic acid with one hydroxyl group.

In the case of chlorogenic acid, it has a more complex structure with two hydroxyl groups bound to the aromatic group and four others are bound to a saturated six-member ring. Probably, some steric restrictions among their hydroxyl groups are responsible for being ready for donation to the free radical [17]. Another reason is that the hydroxyl groups in gallic and procatechuic acids are in *meta* position with respect to the carboxylic group, whereas in vanillic acid the OH is in *para* position with respect to the COOH group.

Many studies about the antioxidant potential of phenolic compounds in fruits or foods have concluded that it is impossible to predict the antioxidant power of a given product by studying just one type of phenolic compound or other kind of antioxidants contained in the product, such as vitamin C or E. In some cases the possible existence of synergistic or antagonistic effects between the various antioxidants present in plant foods and derived products has been discussed [18].

Therefore, we measured the antioxidant activity of individual phenolics and mixes of phenolic acids. Table 1 shows the AOXC of several phenolic acid mixes, representing all possible pair combinations between the four molecules. According to the first combination, between gallic and protocatechuic acid (AB, Table 1-I), where the final concentration of each phenolic was 0.1 mM, the antioxidant arithmetical additive value (47.77 ± 3.1% RSA) was significantly lower (*p* ≤ 0.05) than the experimental AOXC value (67.58 ± 2.8% RSA) determined by DPHH assay. This indicates that there is a synergistic interaction between gallic and protocatechuic acid, where the two antioxidants had a higher AOXC compared to a simple additive contribution of each compound (Figure 1). In a similar way, all other paired combinations showed the same pattern, except AD, which reflected an antagonistic interaction since the arithmetic additive value (36.02 ± 1.3% RSA) was significantly higher (*p* ≤ 0.05) value than the experimental AOXC value (33.03 ± 1.5% RSA). Combinations BD and CD had no significant difference when compared to the arithmetical addition of individual values, and again vanillic acid (D) was involved in such interactions. The chemical structures suggest that the ether group only found in vanillic acid (D) may be related to the lack of hydrogen transfer required for AOXC.

Moreover, triple combinations of phenolic acids are shown in Table 1-II and their AOXC. Three out of four combinations had a synergistic interaction (ACD, ABC, ABD), while only the combination between protocatechuic-chlorogenic-vanillic acid (BCD) had a small antagonistic interaction. Finally, the AOXC of the combination of four phenolic acids is shown in Table 1-III, since these four phenols are found in ‘Ataulfo’ mango pulp. The combination ABCD had an experimental AOXC value of 39.76 ± 2.3% RSA, which is significantly higher (*p* ≤ 0.05) compared to the theoretical additive value calculated as 33.44 ± 1.7% RSA. These results suggest that these four phenolic acids are interacting in a synergic way in mango pulp and probably in other food systems, but this should be tested. 

**Table 1:** Antioxidant capacity of mixtures containing two, three and four phenolic acids. (**A**) gallic acid, (**B**) protocatechuic acid, (**C**) chlorogenic acid, (**D**) vanillic acid.Different letter at each line indicates significant differences (*p* ≤ 0.05).

**I.**	**Individual (0.1 mM)**	**% RSA Real**	**% RSA Theoretical (Sum)**	**Type of Interaction**
	A	30.47 ± 1.8		
	B	17.30 ± 1.3		
	C	13.56 ± 0.8		
	D	5.55 ± 0.2		
	**Combination**			
	AB	67.58µ*a*	47.77µ*b*	Synergic
	AC	44.96µ*a*	44.03µ*b*	Synergic
	BC	34.83µ*a*	30.86µ*b*	Synergic
	AD	33.03µ*a*	36.02µ*b*	Antagonist
	BD	23.66µ*a*	22.85µ*a*	
	CD	20.46µ*a*	19.11µ*a*	
**II.**	**Individual (0.066 mM)**	**% RSA Real**	**% RSA Theoretical (Sum)**	**Type of Interaction**
	A	20.11 ± 1.3		
	B	11.42 ± 0.7		
	C	8.95 ± 0.4		
	D	3.66 ± 0.5		
	**Combination**			
	ACD	58.10µ*a*	32.72µ*b*	Synergic
	ABC	43.03µ*a*	40.48µ*b*	Synergic
	ABD	42.52µ*a*	35.19µ*b*	Synergic
	BCD	19.70µ*a*	24.03µ*b*	Antagonist
**III.**	**Individual (0.05 mM)**	**% RSA Real**	**% RSA Theoretical (Sum)**	**Type of Interaction**
	A	15.23 ± 1.1		
	B	8.65 ± 0.8		
	C	6.78 ± 0.6		
	D	2.77 ± 0.2		
	**Combination**			
	ABCD	39.76µ*a*	33.44µ*b*	Synergic

Only a few studies have focused on the assessment of phenolic interactions in terms of antioxidant activity. Heo *et al*. [13] did not find any synergistic effect between the assayed flavonoids by using the ABTS method and expressing results as a vitamin C equivalent. However, Pinelo *et al*. [19] found an antagonistic effect when phenols interacted at three different temperatures using the DPPH method and several studies showed a synergistic antioxidant effect of flavonoids on free-radical-initiated peroxidation of linoleic acid [20]. 

An antioxidant effect was observed by Pignatelli *et al*. [21] with the flavonoids quercetin and catechin, indicating that these components of red wine act synergistically to inhibit platelet adhesion to collagen and collagen-induced platelet aggregation by virtue of their antioxidant effect.

A common theme in the scientific literature is that interactions between antioxidant molecules do occur, but a mechanism that allows a prediction of synergistic and antagonistic interactions is not apparent. The kind of interaction depends greatly of the specific antioxidants interacting in the system and the condition behind the evaluation [22]. In our case, more than the 80% of our phenolic combinations showed synergistic interactions. Our results suggest that these phenolic acids are capable not only to donate hydrogen atoms to the radical, but they are also able to donate electrons to regenerate other pro-oxidant phenols. This regeneration mechanism maximizes the AOXC of the system to reduce free radicals. According to Leopoldini *et al*. [16], phenolic compounds are capable to transfer electrons to other phenolics or antioxidants, promoting their chemical regeneration. 

In summary, synergistic interactions occurred between the major phenolic acids found in mango ‘Ataulfo’. Based on these results, the importance of choosing the best combination of antioxidants may be advantage when designing new dietary supplements or nutraceuticals. 

## 3. Experimental

Pure commercial standards of gallic, chlorogenic, protocatechuic and vanillic acid were used for all experiments (Sigma-Aldrich, Toluca, Mexico). AOXC was determined by DPPH, and reported as percentages of radical scavenger capacity (RSA). The DPPH assay was conducted according to the method reported by Brand-Williams *et al*. [23] with some modifications. The DPPH solution was adjusted at an absorbance of 1.0 ± 0.02 at 515 nm. Samples of 10 μL were placed in a microplate and 140 μL of DPPH radical were added. After an incubation of 30 min the samples were read at 515 nm using an Omega spectrophotometer (BMG Labtech Inc., Ortenberg, Germany). 

To determine the synergistic or antagonistic interactions between the mango phenolic acids, gallic (A), protocatechuic (B), chlorogenic (C) and vanillic (D) acids were prepared as 0.2 mM concentration stock solutions in 80% methanol. All possible combinations were established. The combinations of phenolic acids were grouped in three sets: combination of two phenolic acids (CB2), combination of three (CB3) and finally the combination of the four phenolic acids (CB4). Each combination was mixed on an equal one mL volume basis maintaining same proportion between the phenolic acids in the mix. The AOXC of each combination was determined using DPPH method as described above. The AOXC of individual phenolic acid at final concentration at each combination were determined to calculate the theoretical value of the mix. This value was established as the sum AOXC values of the individual phenols in each mix. The real AOXC exhibited in each mix was established as the real value. Thus, the theoretical and real values were compared in order to determine if significant synergistic or antagonistic interactions occurred. Results were expressed as means and indicating literals indicate significant differences. Data were statistically analyzed by one-way ANOVA procedure, and the Tukey-Kramer multiple comparison test was used. Standard deviation and variance coefficient between data groups were used to determine significant differences between them at *p* ≤ 0.05 using the statistical software Statgraphics Plus for Windows^®^ v. 5.0. Four replicates were used for each experiment

## 4. Conclusions

Gallic and protocatechuic acid exhibited the highest antioxidant capacity, probably due to their particular chemical conformation and hydroxyl groups content. According to our observations, the phenolic acids present in a mixture can interact, and their interactions can affect the total antioxidant capacity of a solution. It can also be concluded that there are synergistic interactions between the major phenolic acids present in mango ‘Ataulfo’, excluding vanillic acid, which appears to have a negative effect. In the light of the results presented here, the importance of choosing the best combination of antioxidants should be taken in consideration when designing functional foods. More studies with combinations are required in a more mechanistic way, including infrared spectrometry and magnetic nuclear resonance, in order to better understand the mechanisms that are taking place inside an antioxidant system. Also, further studies are needed to evaluate the bio absorption, bioavailability and interactions between these compounds present in mango pulp, after consumption. 
